# Antibiotic resistance, virulence-associated genes analysis and molecular typing of *Klebsiella pneumoniae* strains recovered from clinical samples

**DOI:** 10.1186/s13568-021-01282-w

**Published:** 2021-08-30

**Authors:** Amir Mirzaie, Reza Ranjbar

**Affiliations:** Molecular Biology Research Center, Systems Biology and Poisonings Institute, Baqiyatallah University of Medical Sciences, Tehran, Iran

**Keywords:** *Klebsiella pneumoniae*, Antibiotic resistance, Virulence associated genes, Efflux pumps, Biofilm, Gene expression, Rep-PCR

## Abstract

*Klebsiella pneumoniae* is a multidrug-resistant (MDR) opportunistic pathogen that causes nosocomial infections. Virulence analysis and molecular typing as powerful approaches can provide relevant information on *K. pneumoniae* infection. In the current study, antibiotic resistance, virulence-associated genes analysis, as well as molecular typing of *K. pneumoniae* strains were investigated. Out of 505 clinical samples collected from hospitalized patients, 100 *K. pneumoniae* strains were isolated by standard microbiological methods and subjected to the phenotypic and genotyping analysis. The highest prevalence of resistance was observed against ciprofloxacin (75%), trimethoprim–sulfamethoxazole (73%) and nitrofurantoin (68%). Virulence associated genes including *entB*, *traT*, *ybts, magA, iucC, htrA* and *rmpA* were found in 80%, 62%, 75%, 5%, 30%, 72% and 48%, of the isolates, respectively. The prevalence of biofilm-associated genes including *mrkA, fimH*, and *mrkD* were equally 88% for all tested isolates. Moreover, the efflux pump genes including *AcrAB, TolC* and *mdtK* were observed in 41 (41%), 33 (33%) and 26 (26%) of the strains respectively. A significant statistical association was observed between MDR strains and high expression of efflux pump and biofilm genes. The *K. pneumoniae* strains were differentiated into 11 different genetic patterns using the repetitive element sequence-based PCR (rep-PCR) technique. High prevalence of resistance, presence of various virulence factors, high level of efflux pump, and biofilm gene expression in diverse clones of *K. pneumoniae* strains pose an important health issue in clinical settings.

**Supplementary Information:**

The online version contains supplementary material available at 10.1186/s13568-021-01282-w.

## Introduction

*Klebsiella pneumoniae* is a non-motile Gram-negative bacterium that belongs to the Enterobacteriaceae family (José et al. 2019). *K. pneumoniae* is considered as one of the opportunistic nosocomial pathogens, especially in Iran (Ranjbar et al. 2019). This organism causes a variety of diseases such as bacteremia, pneumonia and urinary tract infection. In recent years, *K. pneumoniae* has attracted the attention of researchers around the world due to its disease severity, resistance against many antibiotics and the difficulty of the treatment (Ranjbar et al. 2016a, b, 2019). Increased multidrug-resistant (MDR) *K. pneumoniae* strains in recent years may be due to overuse and uncontrolled use of antimicrobial agents to treat diseases and infections caused by this bacterium (Fuzi et al. 2020). *K. pneumoniae* has developed several mechanisms for resistance to different antimicrobials (Wanjiang et al. 2019). One of the important mechanisms for developing the MDR is efflux pump systems and biofilm formation capacity (Yoon et al. 2020). Efflux pumps are protein-based structures that are capable to extrude the different toxic substances out of cells (Montazeri et al. 2020). The AcrAB efflux pump system which belongs to the Resistance Nodulation Division (RND) plays an important role in the development of *K. pneumoniae* MDR strains (Xu et al. 2019; Naha et al. 2020; Yoon et al. 2020; Grimsey et al. 2020). The AcrAB-TolC efflux pump is composed of a periplasmic component (AcrA), a transporter located in the inner membrane (AcrB) and an outer membrane compartment (TolC). The AcrAB-TolC efflux pump has a critical role in resistance to multiple antibiotics such as quinolones, tetracycline, and chloramphenicol in MDR strains of *K. pneumoniae* (Shao et al. 2020).

The biofilm-forming ability in *K. pneumoniae* allows the protection of strains from the host immune response and antibiotics in MDR isolates (Sundaramoorthy et al. 2021) and different biofilm-related genes including *mrk* (type 3 fimbriae), *fimH-1* (type 1 fimbrial adhesion) are involved in the biofilm formation (Sahoo et al. 2019; Ranjbar et al. 2019). It was shown that the efflux pumps play an important role in antibiotic resistance and biofilm formation (Tang et al. 2020). Several studies showed a significant correlation between the *K. pneumoniae* antibiotic resistance with efflux pump and biofilm formation ability (Vuotto et al. 2017). Subramanian et al. (2012) found 80% of biofilm-forming isolates from 100 clinical samples showed an MDR phenotype (Subramanian et al. 2012). Several virulence-associated genes include those encoding regulators of mucoid phenotype A (*rmpA*), enterobactin biosynthesis gene (*entB*), outer membrane protein-coding gene (*traT*), yersiniabactin biosynthesis gene (*ybts*), mucoviscosity-associated gene A (*magA*), iron siderophores aerobactin synthase gene (*iucC*) and periplasmic serine endoprotease DegP-like (*htrA*) have a crucial role in the pathogenicity of *K. pneumoniae* strains (Highsmith et al. 1985). Molecular typing of *K. pneumoniae* can be useful in terms of prevention of nosocomial infections in hospitals. (Pasala et al. 2020). Moreover, determination of dominant genotype among isolates can be important for understanding the source of infection and applying prevention procedures (Mukherjee et al. 2019). Various methods including pulsed field gel electrophoresis (PFGE), enterobacterial repetitive intergenic consensus-polymerase chain reaction (ERIC-PCR), randomly amplified polymorphic DNA (RAPD), and repetitive extragenic palindromic polymerase chain reaction (rep-PCR) have been used for molecular typing of *K. pneumoniae* strains. The rep-PCR has been has been successfully used for genotyping of *K. pneumoniae* from various sources. The advantages of rep-PCR over other molecular typing methods include the ability to differentiate between closely related strains of bacteria as well as being a simple, quick, inexpensive, and reliable high-throughput genotyping method (Alharthi et al. 2016).

Due to high prevalence of MDR isolates of *K. pneumonia* in Iran (Heidary et al. 2018; Jafari et al. 2019), the aim of this study was to evaluate the antibiotic resistance profile, distribution of virulence genes, efflux pump and biofilm gene expression, as well as, molecular typing of clinically recovered *K. pneumoniae* strains using rep-PCR.

## Methods

### Bacterial isolates and identification

In this study, a total of 505 clinical samples including blood, urine and cerebrospinal fluid (CSF) were collected from patients admitted to two major hospitals in Tehran, capital of Iran, from January 2018 to July 2019. The *K. pneumoniae* strains were identified using conventional microbiological tests (Collee 2007). The isolated strains were stored at − 20 °C in brain heart infusion broth containing 20% glycerol for further investigation.

### Antimicrobial susceptibility test

The antibiotic susceptibility of *K. pneumoniae* strains was performed based on the Kirby–Bauer disk diffusion method according to the Clinical and Laboratory Standards Institute (CLSI) guidelines (Wayne 2018). The antimicrobial susceptibility assays to 17 antibiotics were performed using commercially available antibiotics including ceftazidime (30 µg), cefotaxime (30 µg), cefoxitin (30 µg), ceftriaxone (30 µg), tobramycin (10 µg), gentamicin (10 µg), streptomycin (30 µg), nalidixic acid (30 µg), ciprofloxacin (5 µg), Imipenem (10 µg), co-trimoxazole (1.25/23.75 µg), chloramphenicol (30 µg), Amoxicillin/Clavulanic acid (AMC, 20/10 µg), meropenem (10 µg), clindamycin (30 µg), polymixin B (10 µg), tetracycline (30 µg) (MAST, Group Ltd., Merseyside, UK). The plates were incubated at 37 °C for 24 h and subsequently, the inhibition zone diameters were recorded in millimeter and interpretation was carried out based on CLSI. *K. pneumoniae* ATCC 13,883 was used as a control in this study. Moreover, multidrug-resistant (MDR) isolates were detected based on their resistance to at least one agent in three or more antimicrobial classes.

### Efflux pump detection

The phenotypic and qualitative detection of the efflux pump in *K. pneumoniae* strains was performed by Cartwheel method (Martins et al. 2011a, b). Briefly, the plates of Muller Hinton Agar culture media containing ethidium bromide were prepared and the culture medium was divided into 8 parts. Then the bacteria with 0.5 McFarland turbidity concentration were streaked on the plates. After 24 h of incubation at 37 °C, the plates were studied under UV transillumination. The strains that had efflux pumps did not show emission of fluorescence (Behdad et al. 2020).

### Biofilm formation test

Phenotypic detection of biofilm formation was performed using Congo red agar test. Briefly, The *K. pneumoniae* strains were cultured in Brain Heart Infusion agar medium enriched with 5% (w/v) sucrose and Congo red based on Freeman et al. (1989). The *K. pneumoniae* strains which formed biofilm exhibited dry dark crystalline colonies and considered exopolysaccharides producers (Hasan et al. 2020).

### Biofilm formation test using quantitative assay

A quantitative biofilm formation test was performed in 96 well tissue culture plates. Briefly, 100 µl of overnight culture with an optical density OD 600 = 0.1 was added into wells. After 24 h incubation, each well was washed twice with PBS and then, stained with crystal violet for 15 min. Finally, the stained cells were dissolved in 33% (v/v) glacial acetic acid and the absorbance was read at 570 nm. The strains were classified upon Donelli et al. 2012 as non-biofilm producers, weak biofilm producers, moderate biofilm former and strong biofilm producers. In addition, the standard strain *K. pneumoniae* ATCC 13,833 and LB broth were considered as a positive and negative control, respectively (Alkhudhairy et al. 2019).

### PCR detection of virulence associated genes

The genomic DNA of each isolate was extracted by DNA extraction kit (Bioneer, Korea) based on to manufacturer’s protocol. The frequency of *entB, trat* and *rmpA* virulence genes, *mdtk, tolC* and *acrAB* efflux pump, *fimH-1*, *mrkA* and *mrkB* biofilm-associated genes were detected by PCR. The PCR conditions were as follow: Initial denaturing at 94 °C for 5 min followed by 30 cycles, each cycle contained 1 min at 94 °C for denaturation, 30 s for annealing (Table 1) and 60 S for extension steps and finally one cycle for the final extension at 72 °C for 10 min. The primer of target genes is given in Table 1.

**Table 1:** The primer sequences of target genes which used in this study

Target gene	Primer sequence (5ʹ to 3ʹ)	Annealing temperature (°C)	References
*acrAB*	F ATCAGCGGCCGGATTGGTAAA R CGGGTTCGGGAAAATAGCGCG	53	Wasfi et al. (2016)
*tolC*	F ATCAGCAACCCCGATCTGCGT R CCGGTGACTTGACGCAGTCCT	51	
*mdtk*	F GCGCTTAACTTCAGCTCA R GATGATAAATCCACACCAGAA	43	
*iucC*	F TGGATTGATGCTCAAACTCTG R TGCATCGCTCATTGACAGTA		Wasfi et al. (2016)
*mrkA*	F ACGTCTCTAACTGCCAGGC R TAGCCCTGTTGTTTGCTGGT	55	Vuotto et al. (2017)
*mrkD*	F CCACCAACTATTCCCTCGAA R ATGGAACCCACATCGACATT	43	Sahly et al. (2008), El Fertas-Aissani et al. (2013)
*fimH*	F GCCAACGTCTACGTTAACCTG R ATATTTCACGGTGCCTGAAAA	43	Wasfi et al. (2016)
*entB*	F CTGCTGGGAAAAGCGATTGTC R AAGGCGACTCAGGAGTGGCTT	49	
*traT*	F GGTGTGGTGCGATGAGCACAG R CACGGTTCAGCCATCCCTGAG	55	El Fertas-Aissani et al. (2013)
*rmpA*	F ACTGGGCTACCTCTGCTTCA R CTTGCATGAGCCATCTTTCA	53	Siu et al. (2011)
*ybtS* *magA*	F GACGGAAACAGCACGGTAAA R GAGCATAATAAGGCGAAAGA F GGTGCTCTTTACATCATTGC R GCAATGGCCATTTGCGTTAG	60	Compain et al. (2014)
*htrA*	F AGAGTTCGCCGTTTTGCCAGGG R ATCAGAGCGCGGATCTTTGCCG	60	Rasheed et al. (2016)
*16 S rRNA*	F AGCCGACCTGAGAGGGTGA R TCTGGACCGTGTCTCAGTTCC	55	Marroquin et al. (2019)

### Gene expression analysis of *acrAB* and *mrkA* gene

Gene expression analysis of *AcrAB* efflux pump and *mrkA* biofilm was performed using quantitative Real-Time PCR method in *K. pneumoniae* strains. Briefly, 100 µl of each bacterial suspension were added into the 96 well plates and incubated for 18 h at 37 °C aerobically. Then, each well was washed using PBS and adherent cells were scraped off using LB broth. Subsequently, the total RNA of collected strains were extracted using an RNA extraction kit (Qiagen, USA) according to instruction protocol. Using cDNA synthesis kit (Fermentase, Lithuania), the purified RNA was converted to complementary DNA (cDNA). In order to perform the Real-Time PCR, each cDNA was used as a template in 20 µl final volume containing 2 µl cDNA, 10 pmol of each primer (Table 1), and 10 µl Power SYBR Green PCR Master Mix (Applied Biosystems) using Bioneer Real-Time PCR equipment (Korea). The *16 S rRNA* was used as a housekeeping gene to normalize the levels of mRNA expression and the relative expression of *AcrAB* efflux pump gene was calculated using ΔΔCт method (Vuotto et al. 2017; Fang et al. 2021).

### Molecular typing of *K. pneumoniae* strains

Molecular typing of *K. pneumoniae* strains were done using the repetitive element sequence-based PCR (rep-PCR) method. The rep-PCR was performed using two following primers: Forward: REP1 5ʹ- III ICG ICG ICA TCI GGC-3ʹ Reverse: REP2 5ʹ- ICG ICT TATCIG GCC TAC-3ʹ as described previously. To study the PCR product, electrophoresis was used on 1.5% agarose gel and the gel was stained with red safe and DNA bands were studied in Geldoc system. Finally, the pattern of DNA bands and their size were examined by Image Lab 4.0 software. The obtaining results were analyzed by gel compare II software using Dice correlation coefficient and the UPGMA (unweighted pair group method with arithmetic mean) method (Hassan and Belal 2016).

### Statistical analysis

All tests used in this study were done in triplicate and the one-way ANOVA test was used for statistical analysis and *p* < 0.05 was considered significant.

## Results

### Isolation and antimicrobial susceptibility pattern of *K. pneumoniae* strains

A total of 100 *K. pneumoniae* strains were isolated from 505 clinical samples. These isolates were recovered from specimens of urine (n = 70), blood (n = 20), Sputum (n = 6) and cerebrospinal fluid (CSF, n = 4) based on routine microbiological methods. Among 100 clinical strains of *K. pneumoniae*, 75 (75%), 73 (73%) and 68 (68%) of strains were resistant to ciprofloxacin, Trimethoprim–sulfamethoxazole and Nitrofurantoin, respectively and 28 (28%), 52 (52%) strains were susceptible to streptomycin and imipenem, respectively (Table 2). In addition, 92 (92%) strains revealed MDR phenotypes and most MDR strains were resistant to imipenem, meropenem and beta-lactam antibiotics. The studied strains were categorized into 25 antimicrobial-resistant patterns (Table 2).

### Phenotypic detection of biofilm formation and efflux pump

**Fig. 1:**
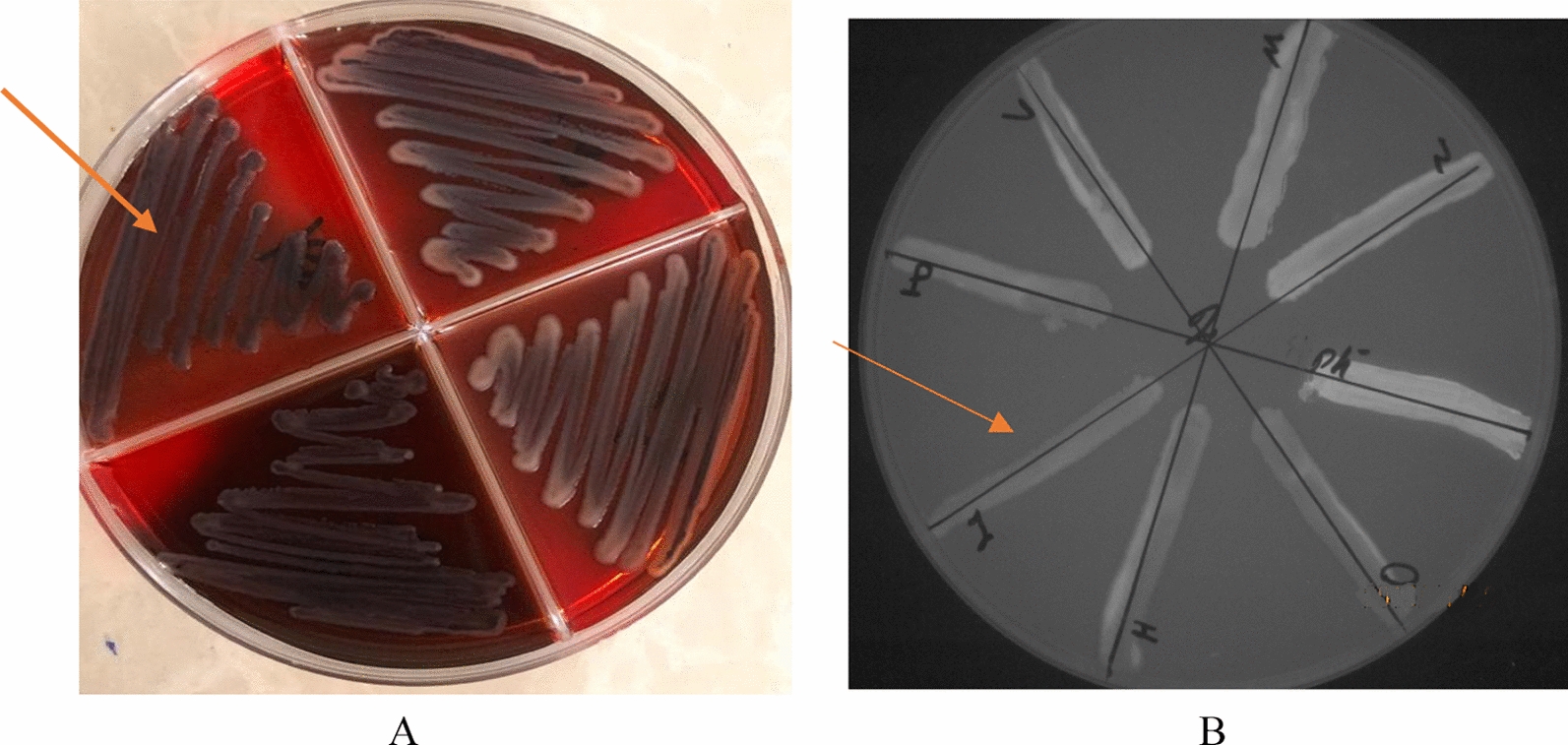
Congo red agar (**A**) and Cartwheel tests (**B**) for detection of biofilm producers and efflux pump system in *K. pneumoniae* strains. As expected, the biofilm forming bacteria exhibited the black appearance in their colonies (Arrow). Moreover, the isolates containing efflux pump did not show fluorescent emission under UV transilluminator (Arrow)

Phenotypic detection of biofilm formation was performed using Congo red agar test and our results showed that 77% (77 strains) of isolates exhibited black colonies, which presumably indicate biofilm formation (Fig. 1A). The other strains formed white colonies which reflecting no biofilm formation. Moreover, the Cartwheel results showed that 90% (90 isolates) of strains had efflux pumps (Fig. 1B).

**Table 2:** Antimicrobial sensitivity, biofilm formation and efflux pump patterns of *K. pneumoniae* strains

Isolates code	Antibiotic resistant pattern	Anti-biotype (AB)	MDR	Biofilm former	Quantitative biofilm formation	Phenotypic efflux pump
K10, K17, K23, K73, K92, K98	CTX, CAZ, CRO, FOX, NA, CP, FM, TE, S, GM	AB1	+	+	2.63 ± 0.14	+
K15, K20	CTX, CAZ, CRO, FOX, NA, CP, FM, TMP	AB2	+	−	0.025 ± 0.03	+
K1, K6, K13, K25	CTX, CAZ, CRO, FOX, TMP, GM, S	AB 3	+	+	2.13 ± 0.19	+
K4, K12, K21, K74, K77, K94	CAZ, FOX, FM, TE, TMP, S	AB 4	+	+	3.52 ± 0.21	+
K18, K35, K49	CTX, CAZ, CRO, FOX, FM, TMP	AB 5	+	+	0.76 ± 0.06	+
K3, K16, K22	CTX, CAZ, CRO, NA, CP	AB 6	+	+	0.38 ± 0.08	+
K8, K45, K75	CTX, CAZ, CRO, FOX, NA, CP, FM, TE	AB 7	+	+	2.69 ± 0.17	+
K5, K41, K76	CTX, CAZ, CRO, FOX, NA, CP, AMC, CF, S, FM, TMP, IMP, GM, MEN	AB 8	+	+	3.4 ± 0.12	+
K19, K31, K72	CTX, CAZ, CRO, NA, CP, FM, TMP, GM, S	AB 9	+	+	1.8 ± 0.07	−
K7	AMC, S, CF, TMP	AB 10	+	+	0.17 ± 0.16	+
K9, K28, K87	FM, CF, AMC, K, GM, TMP, CP, AN, FOX, CTX, CRO, CAZ	AB 11	+	+	0.81 ± 0.007	+
K24, K26, K40	S, CF, AMC, FM, IPM, TMP, MEN, NA, CP	AB 12	+	−	0.16 ± 0.003	+
K14, K39	CL, TE, PB, AN, TMP, GM, K, S, FM, AMC, CF	AB 13	+	+	0.15 ± 0.006	+
K41, K59, K61, K93	AMC, CF, FM, PB, CL, TE	AB 14	+	+	0.94 ± 0.007	−
K36, K43	CP, TE, NA, FM, MEN, TMP, CF, AMC	AB 15	+	+	0.16 ± 0.006	+
K53, K81, K96	CF, AMC, MEN, TMP, IPM, CP, NA	AB 16	+	+	0.20 ± 0.13	+
K27, K30, K62, K88	FM, AMC, CF, IPM, AN, MEN, GM, TMP, CP, TE, NA, CL	AB 17	+	+	0.19 ± 0.001	+
K2, K11, K60, K78, K100	CF, AMC, TMP, CP, TE, NA	AB 18	+	+	0.13 ± 0.006	+
K50, K80, K85, K86	AMC, FM, CF, CP, TE, NA, MEN, IPM, TMP, GM, AN	AB 19	+	+	2.13 ± 0.003	+
K48, K52, K58, K65	AMC, S, CF, FM, IPM, MEN, CP, NA	AB 20	+	+	3.82 ± 0.009	+
K33, K47, K71, K56, K83, K69	CF, K, FM, AMC, GM, MEN, IPM, AN, TMP, NA, TE, CP	AB 21	+	+	0.19 ± 0.007	+
K 34, K 42, K 99	AMC, CF, FM, IMP, MEN, TMP, CP, NA, TE	AB 22	+	+	1.45 ± 0.008	−
K44, K50, K 54, K7, K66, K 79, K 97, K 63, K 64, K 70, K 82	CAZ, CRO, FOX, K, FM, AMC, CF, AN, TMP, GM, IMP, MEN, CP, TE, NA	AB 23	+	−	0.02 ± 0.002	+
K 29, K 32, K 51, K 55, K 57, K 68, K 84	CP, TE, NA, TMP, GM, IMP, MEN, AMC, FM, CF, CAZ, CTX, CRO, FOX	AB 24	+	−	0.029 ± 0.001	+
K 30, K 89, K 90, K 91, K 37, K 38, K 46, K 95	CF, AMC	AB 25	−	+	0.56 ± 0.001	+

*CF* Chloramphenicol, *AMC* Amoxicillin–clavulanic acid, *FOX* Cefoxitin, *CRO* Cefteriaxon, *CTX* Cefotaxime, *CAZ* Ceftazidime, *FM* Nitrofurantoin, *MEN* Meropenem, *IMP* Imipenem, *GM* Gentamycin, *TMP* Trimethoprim–sulfamethoxazole, *NA* Nalidixic acid, *TE* Tetracycline, *CP* Ciprofloxacin, *S* Streptomycin, *K* Kanamycin, *CL* Clindamycin, *PB* Polymixin B, *S* Streptomycin

### Quantitative biofilm production

The results of the quantitative biofilm production test are shown in Table 2 as mean OD 570 values. According to OD values among 77% biofilm producers, 50 isolates (71%) were categorized as strong biofilm former (OD > 0.204), 16 (20%) as moderate biofilm former (0.102 < OD < 0.204) and 11 isolates (14%) as weak biofilm former (0.0551 < OD < 0.102). There is a significant between MDR phenotype, biofilm formation, and efflux pump among *K. pneumoniae* strains (*p* < 0.05). Moreover, there was a significant correlation between biofilm formation in isolates recovered from urine comparing to other type specimens (*p* < 0.05).

### Frequency of biofilm, efflux pump and virulence associated genes

The prevalence of biofilm, efflux pump and virulence-associated genes are given in Additional file 1: Table S1. The *mrkA*, *mrkD* and *fimH* genes encoding type 1 and type 3 fimbrial adhesion engaged in biofilm formation were present in all biofilm former strains. Prevalence of biofilm-associated genes including *mrkA, fimH*, and *mrkD* were equally 88% for all tested. Moreover, the efflux pump genes including *acrAB, tolC* and *mdtK* were observed in 41 (41%), 33 (33%) and 26 (26%) of the strains, respectively. The *acrAB* was more prevalent in *K. pneumoniae* strains comparing to other efflux pump genes. In addition, the *acrAB* efflux pump gene was more prevalent in urine samples in comparison to other clinical specimens. In addition, the virulence-related genes including enterobactin biosynthesis gene (*entB)*, outer membrane protein-coding gene (*traT)*, yersiniabactin biosynthesis gene (*ybts), mucoviscosity-associated gene A (magA*), iron siderophores aerobactin synthase gene *(iucC),periplasmic serine endoprotease DegP-like (htrA)* and mucoid phenotype A *(rmpA)* were found in 80 (80%), 62 (62%), 75 (75%), 5 (5%), 30 (30%), 72 (72%) and 48 (48%), of the isolates, respectively. As reported in Additional file 1: Table S1, analysis of selected genes showed that biofilm was more pronounced among virulence-associated gene-positive than among negative strains. The *entB* virulence gene was detected in all blood, CSF, and sputum isolates. There were 8 virulence profiles (V1–V8) based on detected virulence gene and V1 was the most prevalent virulence type. It should be noted that the virulence genetic profiles showed that the virulence determinants vary between the strains of *K. pneumoniae* that have the same source.

### *acrAB* efflux pump and *mrkA* biofilm gene expression

**Fig. 2:**
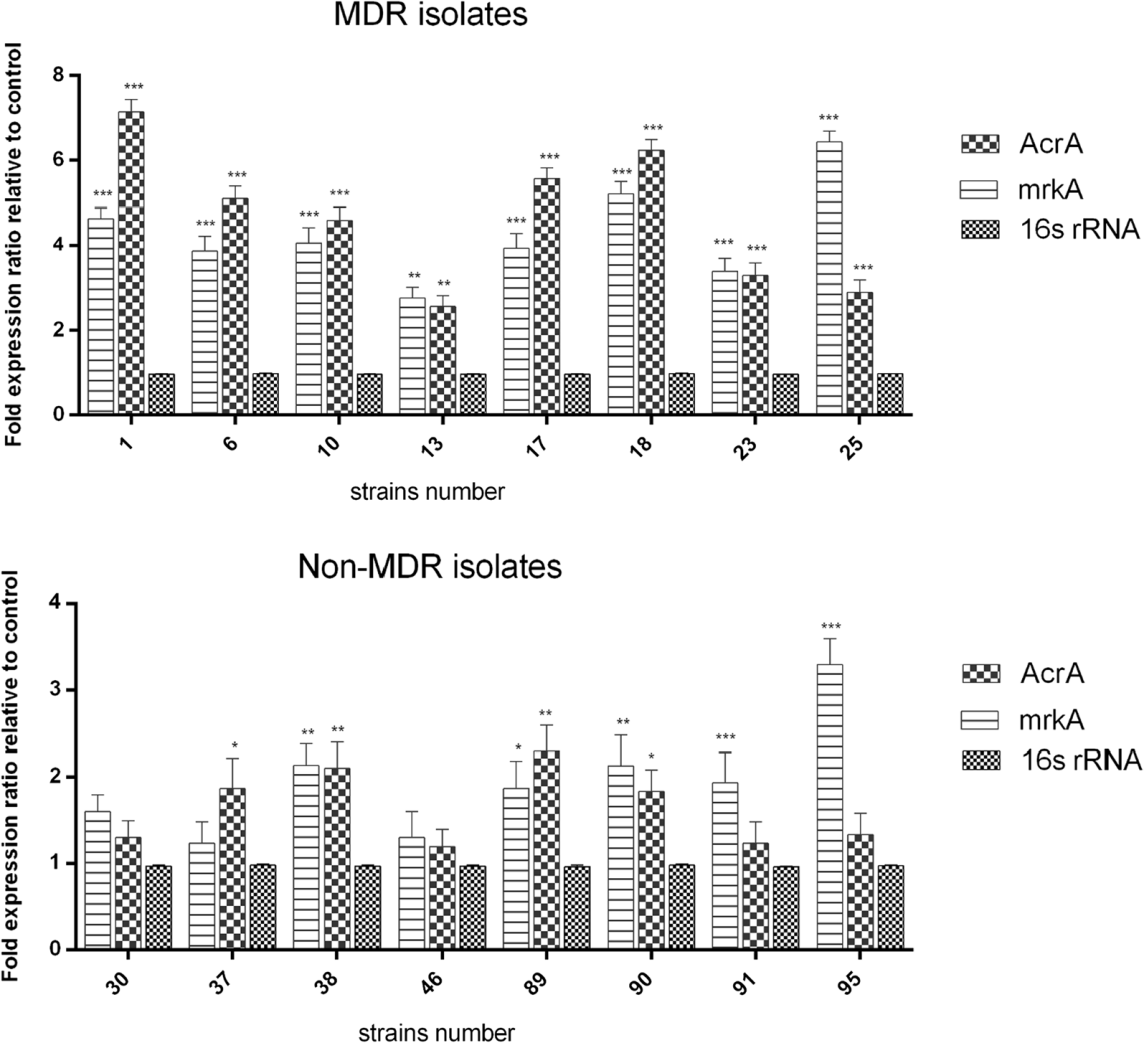
*acrA* and *mrkA* relative gene expression among MDR and non-MDR strains as fold difference between *AcrA, mrkA* gene and *16 S rRNA* gene as a housekeeping gene. ***p < 0.001, **p < 0.01, *p < 0.05

**Fig. 3:**
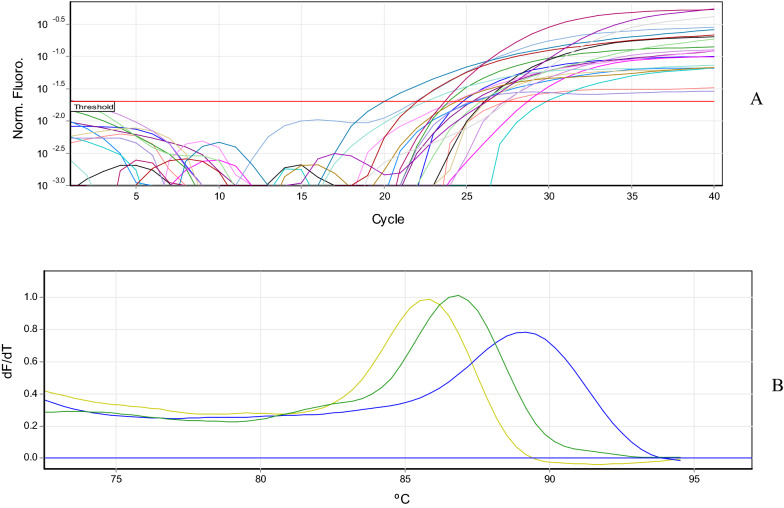
A: Amplification plot of *acrA* and *mrkA* genes, B: melting curve analysis of *acrA, mrkA* and *16 S rRNA* genes. The yellow, green and blue peaks represent the analysis of the melting curve of *acrA* genes with a melting point of 86 °C, *mrkA* gene with a melting point of 87.6 °C and *16 S rRNA* with a melting point of 89 °C

The eight MDR (K1, K6, K10, K13, K17, K18, K23 and K25 isolates) and non-MDR (K30, K37, K38, K46, K89, K90, K91 and K95 isolates) *K. pneumoniae* strains were selected for *acrA* and *mrkA* gene expression analysis. The results of Real-Time PCR showed that *acrA* and *mrkA* genes were up-regulated significantly in MDR isolates comparing to non-MDR isolates (Fig. 2). Amplification plot and melting curve analysis were used to confirm the amplification of the desired genes (Fig. 3A, B).There was a significant relationship between MDR isolates, *acrA*, and *mrkA* gene expression (*p* < 0.05).

### Rep-PCR typing

**Fig. 4:**
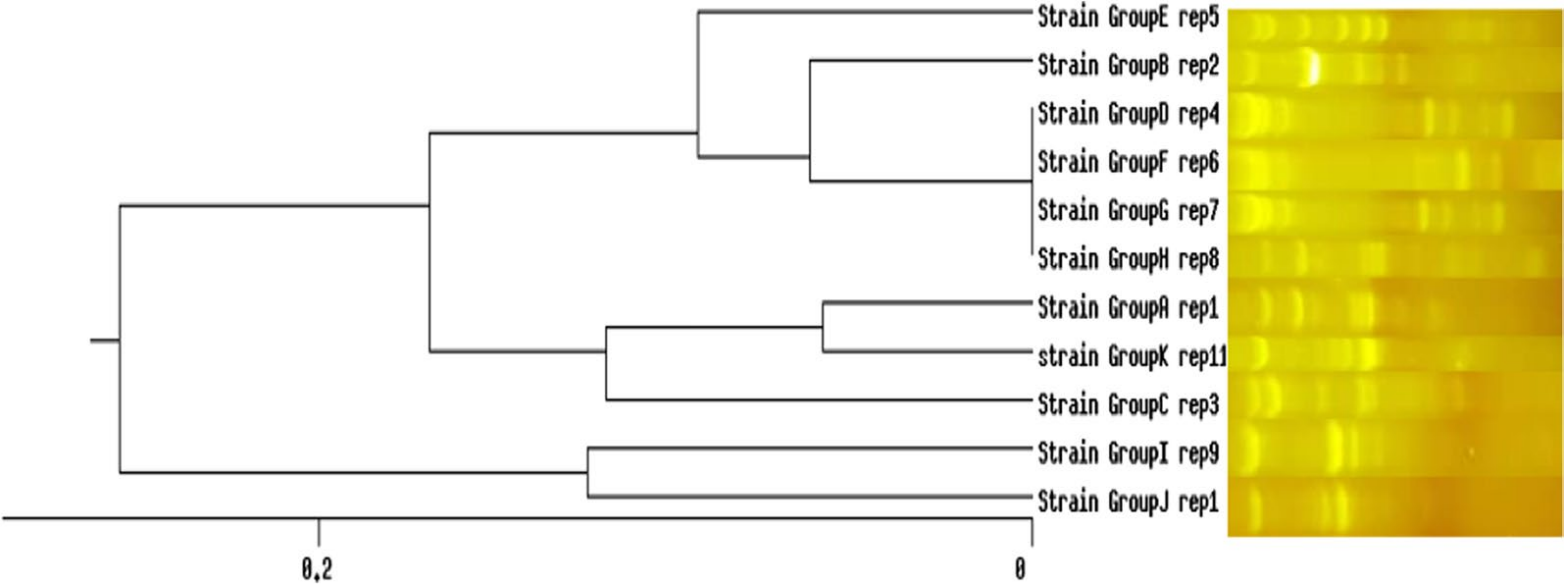
The rep-PCR of representative *K. pneumoniae* strains using the UPGMA based on Dice similarity. Showed 11 rep-types

According to the dendrogram, Repetitive element sequence-based PCR (rep-PCR) revealed 11 distinct patterns of *K. pneumoniae* isolates (Additional file 1: Figure S1 and Fig. 4). The 11 rep genotypes were designed as rep 1 to rep 11. The rep type 4–7 were the most common followed by type rep 1, 9, and 3, which consisted of non-MDR isolates (Group I: K30, K95, K90, K91, Group C: K37, K89, Group J: K38 and K46). The rep type 11 was unique and contained one strain exclusively. Based on statistical correlation tests, the rep type 2, 4, 5, 6 and 7 had significantly correlation with MDR strains and virulence patterns (*p* < 0.05). The strains showed high similarity which may suggest that those isolates consist of a clonal lineage (*p* < 0.05).

## Discussion

Multidrug-resistant (MDR) *K. pneumoniae* strains are amongst the most important cause of several life-treating infections, worldwide (Ghafourian et al. 2011). The extensive use of antimicrobial agents led to a high prevalence of MDR *K. pneumoniae* strains (Ciccozzi et al. 2019). The increasing rate of *K. pneumoniae* strains resistant to multiple antimicrobials is a global public health problem (Khandelwal et al. 2019). In this study, the prevalence of MDR *K. pneumoniae* isolates was 92%. The high rate of MDR strains has been shown in other studies. Manjula et al., indicated that 90.2% of isolates were MDR and the majority of MDR strains were resistant to a high range of antibiotics including penicillin, cephalosporin, fluoroquinolone, aminoglycoside, and sulfonamide (Manjula et al. 2014). Moreover, 48% and 47% of *K. pneumoniae* strains were resistant to imipenem and meropenem, respectively. Yang et al. reported that the highest multidrug resistance rate was detected in human strains of *K. pneumoniae* strains (90.4%). Highest resistance to imipenem 38% (34/89), meropenem 31% (n = 28/89) among *K. pneumoniae* strains was reported by Indrajitha et al. (2021) which is less than the resistance rates seen in our *K. pneumoniae* strains.

From the results, it can be concluded that there has been a significant increase in carbapenem resistant *K. pneumoniae* isolates in Iran. It seems that the production of carbapenemase and metallobetalactamase have an important role in carbapenems resistance (Durante-Mangoni et al. 2019).

One of the possible reasons for the high rate of antimicrobial resistance is the lack of strict policies for use of antibiotics in Iran. Another mechanism of multidrug-resistant is efflux pumps which are used by *K. pneumoniae* strains (Maurya et al. 2019). The efflux pumps could reduce the intracellular concentration of antibiotics which is an important cause of bacterial survival (Xu et al. 2019). In our study, the AcrAB efflux pump was the most common efflux pump in *K. pneumoniae* strains comparing to *mdtk*. It was significantly correlated with MDR phenotype. Our results are consistent with other reports which indicated that the multidrug efflux pump system (AcrAB-TolC) in *K. pneumoniae* strains is responsible for antibiotics especially fluoroquinolones such as ciprofloxacin, tetracycline and beta-lactam antibiotics in MDR isolates. In addition, our results showed that 77% of *K. pneumoniae* strains were able to form biofilm and 89% of biofilm former were MDR. Nirwati et al. showed that 148 (85.63%) of their isolates were biofilm producers, with 45 (26.95%) isolates as strong, 48 (28.74%) isolates as moderate, and 50 (29.94%) isolates as weak biofilm producers. Shadkam et al., using the tissue culture plate assay demonstrated that 75 (75%) of the strains could form a biofilm and 25% of the strains did not have the ability to form a biofilm. The results of Karimi et al. (2021) indicated that Biofilm formation was seen in 62 (75%) of *K. pneumoniae* strains. Strong biofilm formation was observed in 17 (20%) strains and a significant correlation was seen between biofilm formation and antibiotic resistance (*p* < 0.05). Comparison of the results of our study and the other studies indicated that a high percentage of *K. pneumoniae* strains can form biofilms.

Until now, it has been shown that there is a significant correlation between MDR phenotype and the biofilm- forming ability of *K. pneumoniae* strains (Ostria-Hernandez et al. 2018). In addition, the relationship between biofilm formation and antibiotic resistance in *K. pneumoniae* strains at high concentrations, especially sub-inhibitory, has been studied (Maharjan et al. 2018). There was a significant correlation between urine *K. pneumoniae* strains and biofilm formation. Most of the urine- originated strains exhibited strong biofilm capacity.

In the current study, *K. pneumoniae* strains recovered from the clinical samples harbored a high prevalence of efflux pump, biofilm and virulence associated genes. The virulence associated genes were also dominant in MDR strains. Type 1 fimbriae (fimH-1) and Type 3 fimbrial adhesion (*mrkA* and *mrkD*) are the most common bacteria cells adhesive agent which causes *K. pneumoniae* to attach to epithelial and endothelial cells of the urinary tract and causes urinary tract infection (Ranjbar et al. 2007; Panjaitan et al. 2019). Studies have shown that type 3 fimbrial adhesion plays a very important role in biofilm formation in *K. pneumoniae*, but its exact mechanism has not yet been identified (Khalil et al. 2019). Nirwati and colleagues studied the rate of biofilm formation in *K. pneumoniae* strains, and the results of their study showed that 85.63% of the strains constituted biofilm, which was higher than the results of our study (Nirwati et al. 2019). In our study, the *fimH, mrkA* and *mrkD* were detected in all types of urine, blood and CSF isolates. The enterobactin biosynthesis gene (*entB*), serum resistance-associated outer membrane lipoprotein (*traT*) and regulators of mucoid phenotype A (*rmpA*) were detected in 80%, 62% and 48% of *K. pneumonia* strains. The *traT* gene encodes an outer membrane protein which plays an important role in conjugation and inhibition of complement cascade and acts as invasin (Kuş et al. 2017). Wasfi et al. showed that the aerobactin synthase gene (IucC), enterobactin biosynthesis gene (*entB*) and Yersinibactin biosynthesis gene (*Irp-1*) were detected in 32.14%, 85.7% and 28.5% of MDR *K. pneumoniae* isolates, respectively. Ranjbar et al. demonstrated that *fimH-1* (93.04%), *traT* (92.17%), *mrkD* (84.34%), and *entB* (80.86%) were the most commonly detected virulence genes in *K. pneumoniae* strains. Alcántar-Curiel et al. (2013) showed that the *mrkD* adhesin gene and *fimH* were present in 14/69 (20%) and 54/69 (78%) of strains. The results of virulence gene prevalence in various studies have been close to our results, which indicates the high prevalence of virulence genes in *K. pneumoniae* strains. In addition, the results of Real-Time PCR showed that *acrA* and *mrkA* genes were up-regulated significantly in MDR isolates comparing to non-MDR isolates which consistent to another repots. Vuotto et al., Showed that *mrkA* genes was upregulated in biofilm-grown MDR *K. pneumoniae* strains (Vuotto et al. 2017).

Molecular typing is a useful method for the differentiation of nosocomial infections and rep-PCR is a widely used genotyping tool for bacterial strains (Rojas et al. 2017). In our study, out of 100 *K. pneumoniae* strains, rep-PCR differentiated the strains into 11 distinct patterns and most MDR strains were put in the same patterns. Our data confirmed the Lai et al. results which showed pathogenic *K. pneumoniae* strains are heterogeneous, because of variation in genome sequences (Lai et al. 2000). Our results showed the correlation of MDR strains with rep-PCR patterns. However, the rep-PCR revealed no statistically significant correlation with virulence type. In addition, the rep-PCR results showed that the same rep-type in two studied hospitals indicating the same clonal distribution of *K. pneumoniae* in two hospitals. Our findings can be useful for the interpretation of MDR *K. pneumoniae* outbreaks associated with specific patterns in the future. Ghasemian et al. (2017) investigated the rep-PCR analysis for the fingerprinting of *K. pneumonia* isolates. Their results showed that *K. pneumonia* strains placed in 6 clusters and a similarity of 90% was observed among 50–80% of isolates. In Austria in 2010, Grisold et al. investigated a rep-PCR and pulsed field gel electrophoresis (PFGE) methods for the molecular typing of isolated *K. oxytoca*-producing β-lactamase. The results of the automatic rep-PCR method, compared to PFGE, were more accurate for studying the prevalence of isolated *K. oxytoca*-producing β-lactamase with a wider range (Grisold et al. 2010).

In our study, we reported the high prevalence of MDR *K. pneumoniae* strains with resistance to multiple antimicrobial agents, the ability to form biofilm and the presence of efflux pump, biofilm and virulence-associated genes which can be a considered as a major challenge for treatment of *K. pneumoniae* related infections and further spread of resistance genes to other regions. Moreover, high levels of genetic similarity between MDR strains in hospitals showed clonal dissemination of *K. pneumoniae* strains that requires control tools. However, further studies are needed to show other epidemiological aspects of the *K. pneumoniae* strains in our country.

## Supplementary Information

**Additional file 1: Table S1.** Biofilm, efflux pump and virulenceassociation genes among *K. pneumoniae* strains. **Figure S1.**The rep-PCR fingerprints of *K. pneumoniae* strains. Lane 6, 16: 100bp+Ladder. Lane 1: rep 3, Lane 3: rep 9, Lane 4: rep 6, lane 5: negative control,Lane 7: rep 5, Lane 10, 12: rep 7, Lane 11: rep 8, Lane 13: rep 4, lane 15, 18:rep 1, Lane 19, 17: rep 2.

## Data Availability

All data generated or analyzed during this study are included in this manuscript.
