# Supplementary material for: Antibiotic resistance, virulence-associated genes analysis and molecular typing of Klebsiella pneumoniae strains recovered from clinical samples
Source: AMB Express. 2021 Aug 30;11:122. doi: 10.1186/s13568-021-01282-w (PMC8405773; doi:10.1186/s13568-021-01282-w)
Supplement: Supplementary file 1 — Additional file 1: Table S1. Biofilm, efflux pump and virulenceassociation genes among K. pneumoniae strains. Figure S1.The rep-PCR fingerprints of K. pneumoniae strains. Lane 6, 16: 100bp+Ladder. Lane 1: rep 3, Lane 3: rep 9, Lane 4: rep 6, lane 5: negative control,Lane 7: rep 5, Lane 10, 12: rep 7, Lane 11: rep 8, Lane 13: rep 4, lane 15, 18:rep 1, Lane 19, 17: rep 2. [file 13568_2021_1282_MOESM1_ESM.docx]

Table S1. Biofilm, efflux pump and virulence association genes among *K. pneumoniae* strains.

| Isolate code | Biofilm association genes | | | Efflux pump genes | | | Virulence association gene | | |  |  |  |  |  | Virulence gene Type | rep-type | Type of samples |
| --- | --- | --- | --- | --- | --- | --- | --- | --- | --- | --- | --- | --- | --- | --- | --- | --- | --- |
|  | ***mrkA*** | ***mrkD*** | ***fimH*** | ***acrAB*** | ***tolC*** | ***mdtK*** | ***entB*** | ***traT*** | ***rmpA*** | ***ybts*** | ***magA*** | ***iuC*** | ***htrA*** |  |  |  |  |
| **K1** | + | + | + | - | + | - | **+** | **+** | **-** | - | - | - | - |  | V1 | rep-4 Urine | |
| **K2** | + | + | + | + | + | + | **+** | **+** | **+** | + | - | + | + |  | V2 | rep-7 | Urine |
| **K3** | + | + | + | - | + | - | **+** | **+** | **+** | + | - | + | + |  | V2 | rep-2 | Urine |
| **K4** | + | + | + | + | - | - | **+** | **+** | **+** | + | - | + | + |  | V2 | Rep-7 | Urine |
| **K5** | + | + | + | + | - | + | **-** | **+** | **-** | + | - | + | + |  | V3 | rep-4 | Urine |
| **K6** | + | + | + | - | - | + | **+** | **+** | **-** | + | - | - | - |  | V1 | rep-5 | Urine |
| **K 7** | + | + | + | - | + | - | **+** | **-** | **+** | + | - | + | + |  | V4 | rep-11 | Urine |
| **K 8** | + | + | + | + | - | + | **+** | **-** | **+** | + | - | + | + |  | V4 | rep-6 | Urine |
| **K 9** | + | + | + | - | - | - | **-** | **+** | **+** | + | - | + | + |  | V5 | rep-8 | Urine |
| **K 10** | + | + | + | - | + | + | **+** | **+** | **-** | - | - | - | - |  | V1 | rep-2 | Urine |
| **K 11** | + | + | + | + | + | - | **+** | **+** | **-** | - | - | - | - |  | V1 | rep-10 | Urine |
| **K 12** | + | + | + | - | - | - | **+** | **+** | **-** | - | - | - | - |  | V1 | rep-2 | Urine |
| **K 13** | + | + | + | - | + | - | **+** | **+** | **-** | - | - | - | - |  | V1 | rep-10 | Urine |
| **K 14** | + | + | + | - | - | - | **-** | **+** | **-** | + | - | + | + |  | V3 | rep-4 | Urine |
| **K 15** | + | - | - | + | + | - | **+** | **-** | **+** | + | - | + | + |  | V4 | rep-8 | Urine |
| **K 16** | - | + | + | - | - | + | **+** | **+** | **+** | + | - | + | + |  | V2 | rep-5 | Urine |
| **K 17** | + | + | + | - | - | + | **+** | **+** | **+** | + | - | + | + |  | V2 | rep-4 | Urine |
| **K 18** | + | + | + | + | + | + | **+** | **+** | **+** | + | - | + | + |  | V2 | rep-7 | Urine |
| **K 19** | - | + | + | - | - | - | **-** | **+** | **-** | + | - | + | + |  | V3 | rep-2 | Urine |
| **K 20** | + | - | - | + | - | - | **+** | **+** | **-** | - | - | - | - |  | V1 | rep-10 | Urine |
| **K 21** | + | + | + | - | - | - | **+** | **+** | **-** | - | - | - | - |  | V1 | rep-8 | Urine |
| **K 22** | + | + | + | + | + | - | **-** | **+** | **-** | + | - | + | + |  | V3 | rep-4 | Urine |
| **K 23** | + | + | + | - | - | - | **+** | **+** | **-** | - | - | - | - |  | V1 | rep-5 | Urine |
| **K 24** | + | + | + | + | - | - | **-** | **-** | **+** | + | - | + | + |  | V6 | rep-7 | Urine |
| **K 25** | + | + | + | - | + | - | **+** | **+** | **+** | + | - | + | + |  | V2 | rep-2 | Urine |
| **K 26** | + | + | + | - | + | - | **-** | **+** | **+** | + | - | + | + |  | V5 | rep-5 | Urine |
| **K 27** | + | + | + | - | - | - | **-** | **+** | **-** | + | - | + | + |  | V3 | rep-5 | Urine |
| **K 28** | - | + | + | - | + | - | **+** | **+** | **-** | - | - | - | - |  | V1 | rep-11 | Urine |
| **K 29** | - | - | - | + | - | - | **+** | **+** | **+** | + | - | + | + |  | V2 | rep-4 | Urine |
| **K 30** | + | - | - | - | - | - | **-** | **+** | **+** | + | - | + | + |  | V5 | rep-3 | Urine |
| **K 31** | + | + | + | + | - | - | **+** | **+** | **+** | + | - | + | + |  | V2 | rep-7 | Urine |
| **K 32** | + | - | - | + | - | + | **-** | **-** | **-** | + | - | + | + |  | V7 | rep-5 | Urine |
| **K 33** | - | + | + | + | - | + | **+** | **+** | **-** | - | - | - | - |  | V1 | rep-5 | Urine |
| **K 34** | + | + | + | - | + | - | **+** | **+** | **-** | - | - | - | - |  | V1 | rep-10 | Urine |
| **K 35** | - | + | + | - | - | - | **-** | **+** | **-** | + | - | + | + |  | V3 | rep-6 | Urine |
| **K 36** | - | + | + | + | - | - | **+** | **+** | **-** | - | - | - | - |  | V1 | rep-4 | Urine |
| **K 37** | + | - | - | + | - | - | **-** | **+** | **-** | + | - | + | + |  | V3 | rep-1 | Urine |
| **K 38** | + | - | - | + | - | - | **+** | **+** | **-** | - | - | - | - |  | V1 | rep-3 | Urine |
| **K 39** | + | + | + | + | + | - | **+** | **+** | **-** | - | - | - | - |  | V1 | rep-6 | Urine |
| **K 40** | + | + | + | - | - | + | **+** | **-** | **-** | + | - | + | + |  | V8 | rep-5 | Urine |
| **K 41** | + | + | + | - | - | - | **+** | **-** | **+** | + | - | + | + |  | V4 | rep-2 | Urine |
| **K 42** | + | + | + | - | - | - | **+** | **-** | **+** | + | - | + | + |  | V4 | rep-5 | Urine |
| **K 43** | + | + | + | - | - | + | **+** | **-** | **+** | + | - | + | + |  | V4 | rep-7 | Urine |
| **K 44** | + | + | + | + | + | - | **-** | **-** | **+** | + | - | + | + |  | V6 | rep-6 | Urine |
| **K 45** | + | + | + | + | + | - | **+** | **+** | **+** | + | - | + | + |  | V2 | rep-6 | Urine |
| **K 46** | + | + | + | + | + | - | **+** | **+** | **+** | + | - | + | + |  | V2 | rep-9 | Urine |
| **K 47** | + | + | + | - | - | + | **+** | **+** | **-** | - | - | - | - |  | V1 | rep-7 | Urine |
| **K 48** | + | + | + | - | - | - | **+** | **-** | **-** | + | - | + | + |  | V8 | rep-9 | Urine |
| **K 49** | + | + | + | - | + | + | **-** | **-** | **-** | + | - | + | + |  | V7 | rep-6 | Urine |
| **K 50** | + | - | - | + | - | + | **+** | **-** | **+** | + | - | + | + |  | V4 | rep-7 | Urine |
| **K 51** | - | + | + | - | + | - | **+** | **+** | **+** | + | - | + | + |  | V2 | rep-2 | Blood |
| **K 52** | + | + | + | + | + | - | **+** | **+** | **+** | + | - | + | + |  | V2 | rep-5 | Urine |
| **K 53** | + | + | + | + | - | + | **+** | **+** | **+** | + | - | + | + |  | V2 | rep-5 | Sputum |
| **K 54** | + | + | + | - | + | - | **+** | **-** | **+** | + | - | + | + |  | V6 | rep-2 | Blood |
| **K 55** | + | + | + | - | - | - | **+** | **-** | **+** | + | - | + | + |  | V4 | rep-4 | Blood |
| **K 56** | + | + | + | + | - | + | **+** | **-** | **+** | + | - | + | + |  | V4 | rep-5 | Blood |
| **K 57** | + | + | + | - | - | - | **-** | **-** | **+** | + | - | + | + |  | V4 | rep-2 | Urine |
| **K 58** | + | + | + | - | + | + | **+** | **+** | **-** | - | - | - | - |  | V1 | rep-4 | Blood |
| **K 59** | + | + | + | - | - | - | **-** | **+** | **-** | + | - | + | + |  | V3 | rep-7 | Urine |
| **K 60** | + | + | + | - | + | + | **+** | **+** | **-** | - | - | - | - |  | V1 | rep-10 | Urine |
| **K 61** | + | + | + | - | - | - | **+** | **+** | **+** | + | - | + | + |  | V2 | rep-6 | Blood |
| **K 62** | + | + | + | - | - | - | **+** | **+** | **+** | + | - | + | + |  | V2 | rep-5 | CSF |
| **K 63** | + | + | + | - | + | + | **-** | **-** | **+** | + | - | + | + |  | V6 | rep-2 | Urine |
| **K 64** | + | + | + | - | + | + | **+** | **-** | **+** | + | - | + | + |  | V4 | rep-7 | Sputum |
| **K 65** | + | + | + | - | - | - | **+** | **-** | **+** | + | - | + | + |  | V4 | rep-4 | Blood |
| **K 66** | + | + | + | - | - | - | **+** | **-** | **-** | + | - | + | + |  | V8 | rep-5 | Sputum |
| **K 67** | + | + | + | - | + | - | **-** | **-** | **-** | + | - | + | + |  | V7 | rep-7 | Urine |
| **K 68** | + | + | + | - | + | - | **+** | **+** | **-** | - | - | - | - |  | V1 | rep-7 | Blood |
| **K 69** | + | + | + | + | - | - | **+** | **+** | **-** | - | - | - | - |  | V1 | rep-6 | Urine |
| **K 70** | + | + | + | + | - | - | **+** | **+** | **+** | + | - | + | + |  | V2 | rep-5 | Blood |
| **K 71** | + | + | + | + | - | - | **-** | **-** | **-** | + | - | + | + |  | V7 | rep-2 | Urine |
| **K 72** | + | + | + | - | - | - | **+** | **-** | **-** | + | - | + | + |  | V8 | rep-7 | CSF |
| **K 73** | + | + | + | + | - | - | **+** | **-** | **+** | + | - | + | + |  | V4 | rep-6 | Urine |
| **K 74** | + | + | + | - | - | - | **-** | **+** | **-** | - | - | - | - |  | V1 | rep-5 | Urine |
| **K 75** | + | + | + | + | + | - | **+** | **+** | **-** | + | - | + | + |  | V3 | rep-4 | Blood |
| **K 76** | + | + | + | + | + | - | **+** | **-** | **+** | + | - | + | + |  | V4 | rep-6 | Urine |
| **K 77** | - | + | + | - | - | - | **+** | **+** | **-** | - | - | - | - |  | V1 | rep-7 | Blood |
| **K 78** | + | + | + | - | - | - | **+** | **-** | **-** | + | - | + | + |  | V8 | rep-6 | Urine |
| **K 79** | + | + | + | - | - | - | **+** | **-** | **-** | + | - | + | + |  | V8 | rep-2 | Blood |
| **K 80** | + | + | + | + | - | - | **+** | **-** | **-** | + | - | + | + |  | V8 | rep-5 | Blood |
| **K 81** | + | + | + | - | - | - | **+** | **+** | **+** | + | - | + | + |  | V2 | rep-6 | CSF |
| **K 82** | + | + | + | + | - | - | **+** | **-** | **-** | + | + | + | + |  | V8 | rep-4 | Urine |
| **K 83** | + | + | + | - | - | - | **+** | **-** | **-** | + | - | + | + |  | V8 | rep-2 | Blood |
| **K 84** | + | + | + | - | - | - | **+** | **+** | **-** | - | - | - | - |  | V1 | rep-6 | Urine |
| **K 85** | + | + | + | + | - | - | **+** | **-** | **-** | + | - | + | + |  | V8 | rep-2 | Blood |
| **K 86** | - | + | + | + | - | - | **+** | **-** | **-** | + | - | + | + |  | V8 | rep-6 | Sputum |
| **K 87** | + | + | + | - | - | + | **+** | **+** | **-** | - | - | - | - |  | V1 | rep-5 | Blood |
| **K 88** | - | + | + | - | - | + | **+** | **-** | **-** | + | + | + | + |  | V8 | rep-2 | Blood |
| **K 89** | + | - | - | + | - | - | **+** | **-** | **+** | + | - | + | + |  | V4 | rep-9 | Urine |
| **K 90** | + | - | - | + | - | - | **+** | **+** | **+** | + | - | - | + |  | V2 | rep-1 | Sputum |
| **K 91** | + | - | - | + | - | - | **+** | **+** | **+** | + | - | - | + |  | V2 | rep-9 | Urine |
| **K 92** | + | + | + | - | - | - | **+** | **-** | **-** | + | + | + | + |  | V8 | rep-5 | Urine |
| **K 93** | + | + | + | - | - | + | **+** | **-** | **-** | - | + | + | + |  | V8 | rep-2 | Blood |
| **K 94** | - | + | + | - | - | - | **+** | **-** | **+** | + | - | + | + |  | V4 | rep-6 | Urine |
| **K 95** | + | - | - | + | - | - | **+** | **+** | **-** | - | - | - | - |  | V1 | rep-4 | Urine |
| **K 96** | + | + | + | - | + | + | **+** | **+** | **+** | + | - | - | + |  | V2 | rep-2 | Blood |
| **K 97** | + | + | + | + | - | - | **+** | **+** | **-** | - | - | - | - |  | V1 | rep-2 | Urine |
| **K 98** | + | + | + | - | - | - | **+** | **+** | **+** | + | - | - | + |  | V2 | rep-7 | CSF |
| **K 99** | + | + | + | + | - | - | **+** | **+** | **+** | + | - | + | - |  | V2 | rep-5 | Blood |
| **K 100** | + | + | + | - | - | + | **+** | **-** | **-** | + | + | + | - |  | V8 | rep-6 | Sputum |


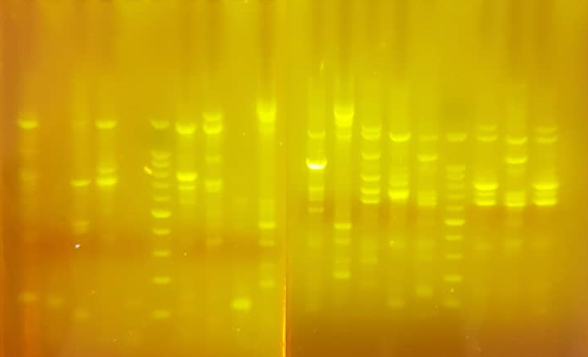

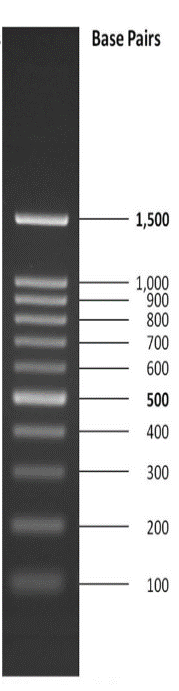


100bp+ Ladder.

1 2 3 4 5 6 7 8 9 10 11 12 13 14 15 16 17 18 19

**Figure S1.** The rep-PCR fingerprints of *K. pneumoniae* strains. Lane 6, 16: 100bp+ Ladder. Lane 1: rep 3, Lane 3: rep 9, Lane 4: rep 6, lane 5: negative control, Lane 7: rep 5, Lane 10, 12: rep 7, Lane 11: rep 8, Lane 13: rep 4, lane 15, 18: rep 1, Lane 19, 17: rep 2.
